# Draft genome sequence of *Levilactobacillus brevis* CHEE98

**DOI:** 10.1128/mra.00993-24

**Published:** 2024-12-19

**Authors:** Ryeong-Hui Kim, DoKyung Lee, Da-Ryung Jung, Amani Sliti, Jae-Ho Shin

**Affiliations:** 1 NGS Core Facility, Kyungpook National University, Daegu, South Korea; 2 Department of Applied Biosciences, Kyungpook National University, Daegu, South Korea; The University of Arizona, Tucson, Arizona, USA

**Keywords:** draft genome sequence, GABA, glutamate decarboxylase, CAI

## Abstract

The draft genome sequence of *Levilactobacillus brevis* CHEE98, isolated from cheese, is presented here. The whole-genome assembly is 2.7 Mbp in size, with a GC content of 45.67%, and includes 2,731 predicted protein-coding genes.

## ANNOUNCEMENT

*Levilactobacillus brevis* plays a crucial role in the fermentation of various foods, including cheese (1). Under anaerobic conditions, *L. brevis* undergoes glycolysis, producing lactic acid and other by-products, which results in a pH decrease during the early stages of fermentation. This drop in pH inhibits the growth of harmful microorganisms and helps stabilize the fermentation environment (2). *L. brevis* is also notable for its ability to synthesize gamma-aminobutyric acid (GABA), a compound associated with various health benefits, such as stress relief and improved sleep quality (3). The production of GABA primarily occurs through the conversion of glutamate via glutamate decarboxylase, a process most active in the acidic environments typical of fermented foods (4).

We performed whole-genome sequencing of *L. brevis* strain CHEE98, isolated from cheese, to identify the genetic elements involved in GABA synthesis and to evaluate their codon usage patterns using the Codon Adaptation Index (CAI).

A total of 0.1 g of mashed Gorgonzola cheese, purchased from a supermarket, was suspended in 0.85% NaCl solution, homogenized, and spread onto De Man–Rogosa–Sharpe (MRS) agar plates, followed by incubation at 30°C for 18 hours. A single colony, selected based on its typical morphology, was isolated and purified by streaking onto a fresh MRS plate. The purified colony was inoculated into fresh MRS broth for genomic DNA extraction using the Wizard Genomic DNA purification kit (Promega, USA). The extracted DNA was measured for quality, size, and quantity using a Nanodrop One Spectrophotometer (Thermo Fisher Scientific, USA), a TapeStation 4150 (Agilent Technologies, USA), and Qubit 3.0 a fluorometer (Thermo Fisher Scientific, USA), respectively.

The DNA, without any additional shearing or size selection, was directly used for library preparation with the Native Barcoding Kit SQK-NBD114 (Oxford Nanopore Technologies) and loaded onto a FLO-MIN114 (R10.4.1) flow cell. Sequencing was performed on the MinION device at the Kyungpook National University NGS core facility in South Korea. FASTQ files with Phred scores of 9+ were generated using Guppy (version 2.24). Filtlong (version 0.2.1) filtered out the bottom 5% of poor-quality reads. After trimming, 454,428,661 bp, 115,161 reads, and an *N*_50_ of 21,749 bp was obtained. *De novo* assembly was performed using Flye (version 2.9.1), and genome annotation was done with the NCBI Prokaryotic Genome Annotation Pipeline (PGAP), both with default parameters (5, 6). The CAI was calculated using the EMBOSS “cai” program (version 6.6.0), with a custom codon usage table derived from the ribosomal proteins of *L. brevis* CHEE98 (7).

The draft genome of *L. brevis* is 2,776,682 bp with 45.67% GC content, consisting of a single linear contig at 56× coverage. CheckM estimated genome completeness at 97.51%. NCBI PGAP annotation revealed 2,754 protein-coding genes, 15 rRNAs, 70 tRNAs, 3 ncRNAs, and 31 pseudogenes (Table 1).

**TABLE 1:** Genetic characteristics of *L. brevis* CHEE98

Feature	Value
Genome size (bp)	2,776,682
Number of contigs	1
G + C ratio (%)	45.67
Total number of genes	2,842
Number of protein-coding genes	2,754
Total number of RNA genes	88
rRNA genes (5S, 16S, 23S)	5, 5, 5
tRNA genes	70
ncRNA genes	3
Pseudogenes	31

Notably, two genes, ACBG88_05690 and ACBG88_08960, encoding glutamate decarboxylase (EC 4.1.1.15)—the key enzyme responsible for the synthesis of GABA from glutamate—were identified. The CAI values of these two genes were then calculated, with ACBG88_05690 and ACBG88_08960 showing CAI values of 0.558 and 0.516, respectively. These results indicate balanced codon usage, suggesting efficient expression.

## Data Availability

The draft genome sequence of *L. brevis* CHEE98 is available in the GenBank database with the accession number CP168078.1. The BioProject number is PRJNA1147898. The raw sequencing data can be accessed with the accession number SRR30409709.
